# Fish Protein Hydrolysates Mitigate the Adverse Effects of No-Fishmeal Diets in Gilthead Seabream Juveniles

**DOI:** 10.1155/anu/1352251

**Published:** 2025-03-07

**Authors:** Cláudia Aragão, Rita Colen, Rita Teodósio, Miguel Cabano, Luís T. Antelo, José Antonio Vázquez, Sofia Engrola

**Affiliations:** ^1^Centro de Ciências do Mar do Algarve, Faro 8005-139, Portugal; ^2^Universidade do Algarve, Faro 8005-139, Portugal; ^3^Biosystems and Bioprocess Engineering Group (Bio2Eng), Marine Research Institute (IIM-CSIC), Vigo 36208, Spain; ^4^Group of Recycling and Valorisation of Waste Materials (REVAL), Marine Research Institute (IIM-CSIC), Vigo 36208, Spain

## Abstract

The aquaculture industry must continue to reduce its reliance on finite marine ingredients and promote biocircularity to enhance sustainability. This study evaluated the effects of no-fishmeal (FM) diets and fish protein hydrolysates (FPHs) on the growth performance, antioxidant status, and immune responses of gilthead seabream (*Sparus aurata*). Following established conditions, two FPHs were prepared from the enzymatic hydrolysis of discards from whole-body blue whiting (*Micromesistius poutassou*) and gurnard heads (*Trigla* spp.); the former contained a higher proportion of large peptides (LPs), while the latter had more small peptides (SPs). Four isoproteic (48%) and isolipic (16%) diets were tested: a commercial (COM)-like diet with 35% FM, 10% poultry meal, and 5% soy protein concentrate; a FUTURE (FUT) diet, without FM or soy protein concentrate, containing 25% poultry meal; and two FUT diets supplemented with FPH (FUTLP and FUTSP). Gilthead seabream (initial weight ± 8.0 g) was distributed into 500 L tanks at an initial density of 1.4 kg m^−3^ and fed the experimental diets to apparent satiety for 8 weeks. Sampling was performed at the end of the growth trial, followed by a digestibility trial. Nutrient and energy digestibilities were significantly lower in the FUT than in the COM diet, with protein and energy digestibilities being 7% and 16% lower, respectively, in the FUT treatment, leading to higher nitrogen losses. Growth performance and feed utilization were negatively impacted in the FUT treatment, with weight gain of only 310 ± 33% compared to 482 ± 22% in the COM treatment. Additionally, immune responses in plasma and antioxidant status in the liver were slightly impaired in the FUT treatment. Supplementation of FPH to the FUT diet mitigated or even reversed these negative effects. The results confirmed that including small- and medium-sized peptides in no-FM diets is more beneficial than using larger ones.

## 1. Introduction

Despite all the alternative and novel ingredients used in contemporary fish feed formulations, fishmeal (FM) remains the benchmark ingredient for aquafeeds. FM is a protein-rich ingredient, with a well-balanced indispensable amino acid profile and valuable micronutrients, including long-chain *n*-3 fatty acids, nucleotides, trace elements, and bioavailable phosphorus. It also has excellent palatability and lacks antinutritional factors [1, 2]. Although the sustainability of FM production has increased over the last decades, with 30% now sourced from fisheries and aquaculture by-products [1], FM inclusion in aquafeeds must be significantly reduced to meet the anticipated growth in aquaculture production.

Future fish diets should aim to reduce or eliminate FM and prioritize sustainability while enhancing food production system resilience. Thus, it is crucial to minimize food-feed competition by maximizing the use of ingredients not used directly for human consumption. Additionally, increasing the use of locally or regionally sourced ingredients can reduce environmental impacts and potentially lower distribution costs. In this context, integrating circular economy principles, such as the valorization of by-products and side streams, is a strategic and resilient approach to developing the aquafeeds of the future.

By-products from terrestrial animal production are widely available in Europe and worldwide [3, 4]. Hence, processed animal proteins (PAPs) resulting from rendering land-farmed animal by-products, such as poultry, feathers, or blood meals, are a valued player within a circular economy [4, 5]. PAPs are often rich protein sources, with a well-balanced indispensable amino acid profile, bioavailable macro- and trace elements, and similar levels of lipid and ash to FM, without antinutritional factors [1, 3, 5]. Therefore, PAPs are competitive in meeting the nutritional needs of farmed fish.

While the potential benefits of using PAPs in fish diets are apparent, research results led to contradictory outcomes. The varying impacts on fish growth and feed utilization may depend on factors such as the source of PAP, the level of FM replacement, and the fish species involved [6–9]. However, even within the same species, inconsistent results have been observed. For example, in gilthead seabream (*Sparus aurata*), Karapanagiotidis et al. [6] reported negative effects on growth and feed utilization as FM replacement levels with poultry meal increased, while Sabbagh et al. [7] found no such effects. In contrast, other studies reported improved growth and feed conversion ratios (FCRs) in gilthead seabream-fed PAP-based diets compared to FM-based diets [8]. Moreover, high levels of FM replacement with poultry meal (50%–100%) did not affect the haematological and blood biochemical parameters of gilthead seabream [6, 7, 9]. Yet, in other species, high levels of FM replacement with PAP have led to adverse effects, such as damage to the architecture of the intestine, gills, liver, and muscle, as well as inducing steatosis in hepatocytes, intestinal inflammation, and bacterial dysbiosis [10–14]. Therefore, further insights into the effect of PAP-based diets on fish physiology are imperative.

The European Union's obligation to land all fishing captures, aimed at gradually eliminating discards (Regulation (EU) No. 1380/2013), requires the development of strategies to upcycle this previously discarded biomass. While producing FM and fish oil from this biomass is one approach to enhancing sustainability, other lower resource strategies, such as the production of fish protein hydrolysates (FPHs), offer significant advantages. FPHs production addresses key issues associated with FM plants, such as high water and energy consumption and the release of organic-rich effluents [15]. Thus, the production and further inclusion of these FPHs in aquafeeds will promote biocircularity in supply chains, contributing to a more sustainable and resource-efficient aquaculture industry.

Studies on FPH inclusion in fish feeds show benefits such as improved growth performance, antioxidant status, immune response, and disease resistance in several fish species (e.g., [16–21]). However, including FPHs at levels above 15%–20% can impair fish growth and feed utilization [22–24]. Additionally, the biological activities of the FPH depend on the molecular weight (MW), amino acid composition, and sequence of the peptides [25]. Low MW peptides are generally more bioactive than large molecular fractions, as they are more easily absorbed in the gastrointestinal tract [25, 26].

Although studies on the effects of future diet formulations on fish growth performance are increasingly available, there is limited information on their impact on fish‘s physiological and immune status. Given the potential benefits of FPHs, it is important to explore their inclusion in future diet formulations to enhance fish robustness. Therefore, this study aims to address these gaps using gilthead seabream, a key species in European aquaculture. Specifically, the objectives are as follows: (1) to assess the effects of no-FM diets, based on ingredients not used directly for human consumption (a future diet formulation named FUT diet), on fish growth performance, antioxidant, and immune status; and (2) to evaluate the benefits of supplementing the FUT diet with FPHs, produced from fisheries by-products, on fish physiological functions.

## 2. Material and Methods

### 2.1. Production of FPHs

Fish discards of blue whiting (*Micromesistius poutassou*) and gurnard (*Trigla* spp.), captured in the North Atlantic Ocean, were separated from commercial species on board, kept in ice, and processed in the port of Marín (Pontevedra, Spain) on the same day of landing. Complete blue whiting specimens were ground and stored at −18°C until further use. Gurnard heads were manually separated from the bodies, ground, and stored at −18°C until processing.

Enzyme hydrolyzes were performed in 5 L glass-reactors (pH-Stat system equipped with additional temperature, agitation, and reagents-addition control), mixing individually 2 kg of fish substrate in 2 L of distilled water (solid:liquid ratio of 1:1), using 5 M NaOH for pH-control. Hydrolysis conditions were based on previous optimal values [15, 27–29]: pH 8.60, 60.6°C, 4 h-hydrolysis, 200 rpm agitation, and 1% enzyme concentration (v:w of substrate). The commercial protease used was Alcalase 2.4 L (2.4–4 AU g^−1^ enzyme, Novozymes A/S, Bagsværd, Denmark).

At the end of the enzymatic digestion process, bones were removed by filtration (100 µm). Gurnard oils were recovered by centrifugation (15,000× *g* for 20 min) and decantation (15 min). For blue whiting, oil recovery was not implemented. In both substrates, final liquid hydrolysates were quickly heated (90°C for 15 min) to denaturate alcalase, freeze-dried for 48–72 h, vacuum-packed, and stored at 4°C. Samples from both FPHs were collected for proximate composition and peptide size distribution analysis (Table S1).

The chemical characterization of dry FPHs was carried out by determining dry matter, ash, and total protein (*N* × 6.25), following standard procedures of the Association of Official Analytical Chemists [30]. Total lipids were analyzed by successive liquid/solid extraction with diethyl ether (in a Soxhlet) at 90°C for 2 h, with final gravimetric quantification by difference. In liquid FPHs, the average MW and number average MW (Mn) of the hydrolysates were analyzed by gel permeation chromatography (GPC-HPLC) using specific Proteema columns (PSS, Mainz, Germany) and a combination of diode array, refractive index, and dual-angle static light scattering detectors [27]. The peptide size distribution of FPHs was analyzed using size exclusion chromatography and UV detection at 220 nm [27]. All analyses were done at least in triplicate.

### 2.2. Experimental Diets

To achieve the study objective, four isoproteic (48%) and isolipidic (16%) diets were formulated (Table 1). The basal diets included a commercial-like diet (named COM diet), which contained 35% FM, 10% poultry meal, and 5% soy protein concentrate, with added commercial FPH; and a FUT diet, without FM and soy protein concentrate and in which poultry meal was increased to 25%. In the FUT diets, different protein hydrolysates were included: feather meal hydrolysate and hemoglobin powder (FUT diet), FPH from gurnard heads with a higher proportion of large peptides (LPs) (FUTLP diet), and FPH from whole-body blue whiting with a higher proportion of small peptides (SPs) (FUTSP diet). These FPHs were included to ensure an equivalent dietary protein contribution.

The diets, with a pellet size of 1.5–2.0 mm, were manufactured by extrusion at SPAROS Lda. (Olhão, Portugal), following the procedures described by Aragão et al. [31], and were stored in a cool, aerated room. Diet samples were collected for analysis of proximate composition (Table 1) and amino acid content (Table S2), as described in Section 2.6.1.

### 2.3. Growth Trial

Gilthead seabream (*S. aurata*) juveniles (± 5.0 g) were acquired from commercial aquaculture and transported to the CCMAR facilities at Ramalhete Experimental Research Station (Faro, Portugal). The fish were acclimated to the new facilities in a flow-through system with aeration and were fed to apparent satiety with a commercial diet (NEOGOLD Blue, Aquasoja, Sorgal, S.A., Portugal; 58% crude protein [CP], 14% crude fat [CF]) for 2 weeks.

The experiments were conducted according to the European and Portuguese legislation on the protection of animals used for scientific purposes (Directive 2010/63/EU of the European Parliament and the Council of the European Union and Decreto-Lei n° 113/2013 de 7 de Agosto, respectively) and were directed by trained scientists following FELASA category C recommendations.

Twelve homogenous groups of 90 juvenile gilthead seabream (initial weight: 8.05 ± 0.50 g) were distributed in weighed groups of 10–20 fish into 500 L tanks, with an initial density of 1.4 kg m^−3^. Fish were supplied with flow-through aerated seawater (temperature: 21.7 ± 1.6°C; salinity: 37.3 ± 0.4‰; dissolved oxygen in water above 85% of saturation) and subjected to natural photoperiod changes from mid-May to mid-July. Each diet was randomly assigned to triplicate tanks and tested over 8 weeks. Fish were fed to apparent satiety by hand three times a day from Monday to Saturday and twice on Sundays. Fish mortality and apparent feed intake were recorded daily, with utmost care taken to avoid feed losses.

At the beginning of the trial, 15 fish were euthanized (1.5 mL L^−1^ 2-phenoxyethanol; Sigma–Aldrich, Spain), pooled, and frozen at −20°C for proximate composition analysis, as described in Section 2.6.1. At the end of the trial, 18 fish per tank were individually measured and weighed under mild anesthesia (0.3 mL L^−1^ 2-phenoxyethanol). Of these, three fish per tank were euthanized, pooled, and frozen at −20°C for proximate composition analysis. Blood was collected from the caudal vein of six fish using heparinized syringes, and plasma samples were collected for analysis of immune response indicators in individual fish, as described in Section 2.6.2. The fish were then euthanized, liver and perivisceral fat were collected and weighed, and the liver was frozen at −80°C for later analysis of antioxidant status indicators in individual fish, as described in Section 2.6.3. The remaining fish in each tank were bulk-weighed and counted. All samples were collected after a 24-h fasting period.

### 2.4. Key Performance Indicators

Growth performance, feed utilization, and nutrient retention indicators were calculated using the following formulas:

Weight gain (%) = 100 × (final body weight − initial body weight) × initial body weight^−1^.

Daily growth index (DGI) = 100 × (final body weight^1/3^ − initial body weight^1/3^) × days^−1^.

Condition factor (*K*) = 100 × body weight × total length^−3^.

Hepatosomatic index (HSI, %) = 100 × liver weight × body weight^−1^.

Perivisceral fat index (PFI, %) = 100 × perivisceral fat weight × body weight^−1^.

Survival (%) = 100 × final number of fish × initial number of fish^−1^.

Daily voluntary feed intake (VFI, % day^−1^) = 100 × apparent feed intake × ABW^−1^ × days^−1^, where ABW is average body weight = (final body weight + initial body weight)/2.

FCR: apparent feed intake × weight gain^−1^, where weight gain = final fish weight − initial fish weight.

Protein efficiency ratio (PER) = weight gain × CP intake^−1^.

Protein, lipid, or energy retention (PR, LR, or ER, %) = 100 × (final whole-body protein, lipid, or energy content − initial whole-body protein, lipid, or energy content) × (CP, crude lipid, or gross energy intake)^−1^.

Nitrogen (N) gain (mg N kg^−1^ day^−1^) = (final whole-body N content − initial whole-body N content) × ABW^−1^ × days^−1^.

Faecal N losses (mg N kg^−1^ day^−1^) = Crude N intake × apparent digestibility coefficient (ADC) (%) of N.

Metabolic N losses (mg N kg^−1^ day^−1^) = crude N intake − N gain − faecal N losses.

### 2.5. Diet Digestibility

At the end of the growth trial, 12 groups of 16 gilthead seabream juveniles (initial body weight: ± 67 g) originating from the growth trial were stocked in 100 L cylinder-conical tanks (temperature: 22.3 ± 1.2°C; salinity: 36.9 ± 2.5‰). The outlet water from these tanks ran through a recipient adapted as a faeces-settling decantation system. The ADCs of the dietary components were determined by the indirect method, using yttrium oxide as an inert tracer.

Triplicate groups of fish were allocated to the same experimental diet used during the growth trial and were hand-fed to apparent satiety twice daily. Fish were allowed to adapt to the new conditions for 10 days before starting faeces collection. Once faeces collection began, tanks were thoroughly cleaned after the second feeding to remove any uneaten feed. Faecal samples were collected daily before feeding, for an average of 12 days. Pooled faeces samples per tank were frozen at −20°C until analysis of proximate composition and yttrium content, as described in Section 2.6.1.

The following formulas were used to calculate the ADC of the dry matter and nutrients or energy, respectively [32]:

  
$$\begin{array}{c} \text{ADC}\left(\%\right)=100\times \left[1-\frac{{\text{ dietary Y}}_{2}{\text{O}}_{3}\text{ level}}{{\text{faecal Y}}_{2}{\text{O}}_{3}\text{ level}}\right], \end{array}$$

  
$$\begin{array}{c} \text{ADC}\left(\%\right)=100\times \left[1-\frac{{\text{ dietary Y}}_{2}{\text{O}}_{3}\text{ level}}{{\text{faecal Y}}_{2}{\text{O}}_{3}\text{ level}}\times \frac{\text{faecal nutrient or energy level}}{\text{dietary nutrient or energy level}}\right]. \end{array}$$

### 2.6. Analytical Methods

#### 2.6.1. Chemical Analyses

Dry matter, ash, protein, lipids, and energy were analyzed in duplicate for diet, pooled fish, or faeces samples, following the standard procedures of the Association of Official Analytical Chemists [30]: dry matter by drying the samples at 105°C for 24 h; ash content by incineration in a muffle furnace at 550°C for 6 h; CP (*N* × 6.25) using a Leco nitrogen analyzer (Model FP- 528; Leco Corporation, St. Joseph, USA); crude lipids by petroleum ether extraction using a Soxtherm Multistat/SX PC (Gerhardt, Germany); gross energy by combustion in an adiabatic bomb calorimeter (Werke C2000; IKA, Staufen, Germany) calibrated with benzoic acid.

Phosphorus in diets and yttrium in diet and faeces were analyzed after sample digestion with 69% nitric acid (v:v) in a high-performance microwave digestion unit (Discovery SP-D, CEM Corporation). Samples were then read using microwave plasma-atomic emission spectroscopy (MP-AES, Model 4200, Agilent), along with calibration standards and blanks.

The total amino acid content in diets was determined on hydrolyzed samples (6 M HCl at 116°C for 48 h in N-flushed glass vials), followed by precolumn derivatisation with Waters AccQ Fluor Reagent (6-aminoquinolyl-N-hydroxysuccinimidyl carbamate). Analyses were performed by ultra-high-performance liquid chromatography (UPLC) in a waters reversed-phase amino acid analysis system, using the AccQ Tag method (Waters, USA) with norvaline as the internal standard [8].

#### 2.6.2. Immune Status

The immune status of the fish was assessed in individual plasma samples (*n* = 6 per tank, *n* = 18 per treatment), as described by Machado et al. [33]. All assays were performed spectrophotometrically in 96-well flat-bottom microplates using a temperature-controlled microplate reader (Synergy 4 BioTek, Winooski, VT, USA).

Briefly, alternative complement pathway (ACH50) activity in plasma was measured following the procedure described by Oriol Sunyer and Tort [34], using sequentially washed rabbit red blood cells (Probiologica Lda., Portugal) and serially diluted plasma. The ACH50 units were defined as the concentration of plasma inducing 50% hemolysis of rabbit red blood cells after 100 min of incubation at room temperature.

Total peroxidase activity in plasma was analyzed according to the procedure described by Quade and Roth [35], by measuring the color change reaction with 3,3,5,5-tetramethylbenzidine (TMB) hydrochloride (Sigma–Aldrich) and H_2_O_2_. Total peroxidase activity was expressed as IU mL^−1^ plasma, with one unit of peroxidase defined as the amount producing an one optical density change in absorbance.

#### 2.6.3. Antioxidant Status

The antioxidant status of the fish was assessed by measuring lipid peroxidation (LPO), total antioxidant capacity (TAC), catalase (CAT) activity, and total glutathione (GSH) content in individual liver samples (*n* = 6 per tank, *n* = 18 per treatment), following the procedures described by Xavier et al. [36].

Briefly, liver samples were homogenized in K-phosphate buffer (0.2 M, pH 7.4) using a TissueLyser (Star-Beater, VWR, Radnor, PA, USA). An aliquot was preserved with 4% butylated hydroxytoluene (BHT) (Sigma–Aldrich) in methanol, for endogenous LPO determination. The other aliquot was centrifuged (20 min at 10,000× *g*, 4°C), and the postmitochondrial supernatant was used for TAC, CAT, and GSH analysis. All determinations were performed spectrophotometrically in 96-well flat-bottom microplates using a temperature-controlled microplate reader (Synergy 4 BioTek, Winooski, VT, USA). Protein concentration was determined by the Bradford method [37], using bovine serum albumin as a standard.

LPO was determined spectrophotometrically by measuring thiobarbituric acid-reactive substances (TBARSs) [38]. Samples were read at 535 nm, and results were expressed in nmol TBARS per mg protein.

TAC was assessed using the colored 2,2-azino-bis-(3-ethylbenzothiazoline-6-sulfonic acid) radical cation (ABTS^+^). The reduction of ABTS^+^ to its colorless original molecule (ABTS) was measured as a change in absorbance at 660 nm, and the reaction rate was calibrated with Trolox [39]. Results were expressed in mmol Trolox equivalent per mg protein.

CAT activity was determined by measuring the decrease of H_2_O_2_ concentration at 240 nm [40]. Results were expressed in IU per mg protein, with one IU defined as the amount of CAT required to decompose 1 *µ*M of H_2_O_2_ per minute at pH 7.0 and 25°C.

Total glutathione content was determined at 412 nm using a recycling reaction of reduced glutathione with 5,5′-dithiobis-(2-nitrobenzoic) (DTNB) acid (Sigma–Aldrich) in the presence of excess glutathione reductase. GSH content was calibrated using reduced glutathione as a standard [41]. Results were expressed in mmol of GSH per mg protein.

### 2.7. Statistical Analysis

All the results are presented as means ± standard deviation (SD). Data expressed as percentages were transformed (arcsine square root) before statistical analysis [42]. Data were checked for normality and homoscedasticity using the Shapiro–Wilk and Levene's tests. One-way ANOVA followed by Tukey's HSD test was used if the data met the assumptions of normality and homoscedasticity; otherwise, the Kruskal–Wallis test was applied. The significance level was set as *p* < 0.05. The statistical analysis was conducted using the Statistica 8.0 software (StatSoft. Inc., USA).

## 3. Results

### 3.1. Diet Digestibility

The ADCs of dry matter, protein, lipids, and energy were significantly lower in the FUT than in the COM diet (Table 2). The inclusion of FPHs in the FUT diet led to intermediate ADC values for dry matter, lipids, and energy, with no significant differences observed between the COM and the FUTLP diets. The ADC of protein was similar in the COM, FUTLP, and FUTSP diets, and significantly higher than in the FUT diet.

### 3.2. Fish Growth Performance and Feed Utilization

Growth performance (final weight, weight gain, and DGI) and *K* were significantly lower in fish fed the FUT diet compared to the COM-fed fish (Table 3). The FUTLP and FUTSP diets improved the fish growth compared to the FUT diet, with no significant differences in weight gain, DGI, and *K* between fish fed the FUTSP and COM diets. The HSI was unaffected by the dietary treatment. The PFI was only affected by the major dietary protein source, thus fish-fed FUT diets presented lower PFI than the COM-fed fish, regardless of FPH supplementation.

The VFI was unaffected by the dietary treatments (Table 4). The FCR and PER values were negatively affected in fish fed the FUT compared to the COM diet. However, FPH inclusion mitigated (FUTLP) or reversed (FUTSP) these effects. The fish whole-body composition was not significantly different among treatments, except for higher protein content in the FUT treatment (Table 5). The retention efficiency of protein and lipids was similar across dietary treatments (Table 4). ER was significantly lower in fish fed the FUT compared to the COM and FUTSP diets, with fish fed the FUTLP diet presenting intermediate values.

**Figure 1:**
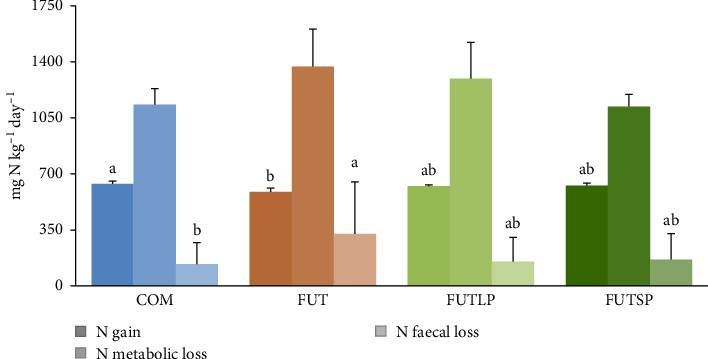
Daily nitrogen (N) balance in gilthead seabream juveniles fed a commercial (COM) or a future (FUT) diet containing fish protein hydrolysates with large (FUTLP) or small (FUTSP) peptides for 8 weeks. Values are presented as means ± standard deviation (*n* = 3). Different letters indicate significant differences (*p* < 0.05) among treatments. The absence of letters indicates no significant differences.

Concerning the daily N balance (Figure 1), fish fed the FUT diet had significantly lower N gain and higher faecal losses compared to those fed the COM diet. Fish fed the FUTLP and FUTSP diets presented intermediate values for both N gain and faecal losses. The N metabolic losses were not affected by the dietary treatments.

### 3.3. Immune and Antioxidant Status

**Figure 2:**
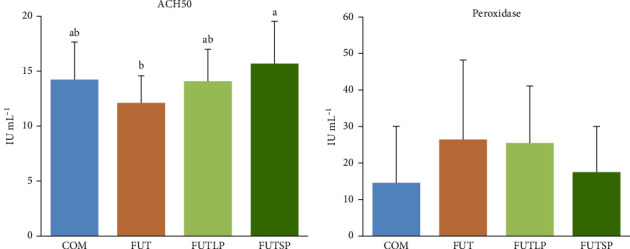
Alternative complement pathway (ACH50) and peroxidase activities in the plasma of gilthead seabream juveniles fed a commercial (COM) or a future (FUT) diet containing fish protein hydrolysates with large (FUTLP) or small (FUTSP) peptides for 8 weeks. Values are presented as means ± standard deviation (*n* = 18). Different letters indicate significant differences (*p* < 0.05) among treatments. The absence of letters indicates no significant differences.

Plasma ACH50 activity was significantly lower in fish fed the FUT diet compared to those fed the FUTSP diet, with fish from the COM and FUTLP diets presenting intermediate values (Figure 2). Peroxidase activity in plasma did not differ significantly among the dietary treatments.

**Figure 3:**
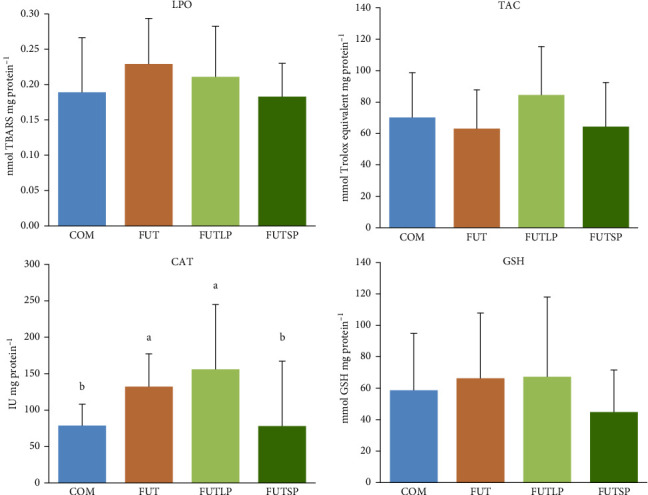
Lipid peroxidation (LPO), total antioxidant capacity (TAC), catalase (CAT) activity, and total glutathione (GSH) content in the liver of gilthead seabream juveniles fed a commercial (COM) or a future (FUT) diet containing fish protein hydrolysates with large (FUTLP) or small (FUTSP) peptides for 8 weeks. Values are presented as means ± standard deviation (*n* = 18). Different letters indicate significant differences (*p* < 0.05) among treatments. The absence of letters indicates no significant differences.

The biomarkers of antioxidant status were not significantly affected by the dietary treatment, except for the CAT activity (Figure 3). The hepatic activity of this enzyme was significantly higher in fish fed the FUT and FUTLP diets compared to those fed the COM and FUTSP diets. The latter were not significantly different.

## 4. Discussion

Aquaculture is vital for ensuring food security for a growing population that demands more aquatic foods. Sustainability is a priority to secure growth at a pacing rate, and formulating future aquafeeds that respect and promote environmentally sound practices is paramount. In this study, no-FM diets based on poultry meal and FPHs produced from fisheries by-products were tested. This approach aimed to enhance the use of by-products from the food production system, improving aquaculture circularity and sustainability.

Marine ingredients in aquaculture diets typically improve feed acceptability and intake, balancing out less palatable ingredients [1]. For instance, gilthead seabream-fed poultry meal-based diets without FM showed reduced feed intake, even with FPH and squid meal added as feeding attractants [6, 43]. Notably, no impacts on feed palatability were observed in this study. Including either FPH or a mixture of feather meal hydrolysate and hemoglobin powder in no-FM diets resulted in similar fish feed intake to the COM treatment, which contained significant levels of marine ingredients. Thresholds for the inclusion of blood and feather meals have been reported around 10%, as higher levels negatively impact feed intake [1]. Additionally, dietary inclusion levels of FPHs in the present study were nearly 10%, higher than that used by Randazzo et al. [43]. Thus, the inclusion levels of the hydrolysates used were within optimal values and did not impair feed intake.

Despite the positive effect on feed intake, PAPs might have impaired diet digestibility. For instance, in Atlantic salmon (*Salmo salar*), ADCs of N and lipids were significantly reduced when FM was replaced by a mixture of poultry and porcine blood meals [44]. Similarly, the FUT diet in this study presented lower nutrient and energy digestibility than the COM diet, resulting in increased environmental impact due to higher N faecal losses. Likewise, in European seabass (*Dicentrarchus labrax*), adding 3% of porcine blood hydrolysate to plant-based diets reduced nutrient and energy digestibility, leading to increased N faecal losses [45] and increasing environmental impact.

The inclusion of FPHs in FM and plant-based diets increased dietary protein and dry matter digestibility in several fish species [19, 20, 23, 46, 47]. Likewise, in this study, the inclusion of FPHs partially reversed the negative effects on diet digestibility, thus contributing to lower N faecal losses. FPHs have been shown to increase the expression of intestinal peptide and amino acid transporters, mitigating the adverse effects of plant-based diets [46, 48]. These enhanced transport may explain the increased protein digestibility observed in the FUTLP and FUTSP diets compared to the FUT treatment. Additionally, dietary FPH inclusion in poultry meal-based diets has been shown to repair intestinal mucosal barrier damage and restore gut homeostasis [12], thus maintaining gut health and minimizing environmental impacts.

The lower digestibility of the FUT diet negatively affected gilthead seabream growth performance and FCR. These negative results contrast with previous studies on gilthead seabream-fed low- or no-FM diets, where no or even positive effects of dietary poultry meal inclusion were observed [7, 8, 43]. These negative effects may be related to the lower diet digestibility, likely resulting from the inclusion levels of feather meal hydrolysate and/or porcine hemoglobin powder. Previous studies showed that the digestibility of low-FM diets for gilthead seabream juveniles was not affected by a lower level of feather meal hydrolysate addition (4%), even with higher poultry meal inclusion [8].

The inclusion of both FPHs partially reversed the negative effects on gilthead seabream growth and feed utilization. Similarly, Pham et al. [13] reported that the negative effects of a poultry meal-based diet on the growth performance and feed efficiency of pompano (*Trachinotus blochii*) could be partially mitigated by FPH inclusion. Moreover, FPHs have been able to reverse the negative impacts on growth performance in fish-fed plant-based diets [18, 47, 49]. Notably, the growth performance and feed utilization of gilthead seabream fed the FUTSP diet were not significantly different from those fed the COM diet. This result corroborates previous studies that found a positive effect on the growth and feed utilization of fish-fed diets with FPHs containing small MW peptides (<1.0 kDa) compared to larger MW peptides [19, 50].

Most studies report no effects of replacing FM with poultry meal on the muscle or carcass macronutrient composition of gilthead seabream juveniles [7–9] and other fish species [11, 51–53]. However, this study found that feeding gilthead seabream the FUT diet increased whole-body protein composition compared to those fed the COM diet. In contrast, previous studies have shown a decrease in whole-body protein composition in pompano and cobia (*Rachycentron canadum*) when FM was replaced with poultry meal [13, 54]. Notably, as observed in the study by Pham et al. [13], the inclusion of FPH in PAP-based diets reversed the alterations in protein content. Additionally, similar to findings in Atlantic salmon, the negative impacts of PAP on diet digestibility reduced the retention efficiency of energy [44]. However, further inclusion of FPHs in the FUT diet mitigated or even reversed these negative effects.

The HSI was not affected by the dietary treatments, consistent with previous studies reporting no relation between dietary poultry meal inclusion and HSI values in gilthead seabream juveniles [6, 7, 9]. However, this effect seems to be species dependent. In other fish species, increasing levels of FM replacement by poultry meal have been associated with either an increase [11, 13, 53, 54] or no effect [51, 52] on HSI. Conversely, the PFI was directly related to the dietary protein source, with lower values observed in gilthead seabream-fed FUT diets, regardless of FPH supplementation. This result contrasts with findings by Zhou et al. [10] in hybrid grouper (*Epinephelus fuscoguttatus* × *Epinephelus lanceolatus*), which reported an increase in PFI with increasing levels of FM replacement by poultry meal. Similarly, barramundi (*Lates calcarifer*)-fed poultry meal-based diets supplemented with FPH had a similar intraperitoneal fat index to those fed FM-based diets [17]. Therefore, the effect of FPH on PFI deserves further attention, particularly in species where this index may be significantly increased, such as gilthead seabream and European seabass, as it can influence consumer perception.

Few studies have analyzed the effects of replacing FM with PAPs on fish immune status, though some evidence suggests slight impairments. High levels of PAPs in the diet triggered an inflammatory response in hybrid grouper [11], and a significant decrease in lysozyme activity was observed in barramundi-fed no-FM diets based on poultry meal [14]. In this study, a slight impairment in the immune response of gilthead seabream was observed due to the change in dietary protein source, with some immunomodulatory effects due to FPH supplementation.

While the lower values of ACH50 activity found in the FUT treatment were only numerically lower than those in the COM treatment, gilthead seabream fed the FUTSP diet showed significantly higher activity, suggesting an enhancement of the fish‘s immune status. Likewise, appropriate levels of FPH supplementation increased lysozyme and complement activities or gene expression in fish-fed FM-based diets [24, 55, 56]. For example, in European seabass, FPH supplementation mitigated the negative effects on lysozyme activities caused by plant-based diets, although it did not affect ACH50 and peroxidase activities [18]. Conversely, no significant effect of FPHs on these immune biomarkers was found in barramundi-fed FM or poultry meal-based diets [17, 57]. These varying responses in the immune biomarkers could be attributed to differences in species, dietary protein sources, or the MW of peptides in the FPH. The enhanced immune response observed in gilthead seabream fed the FUTSP diet supports previous reports that FPHs containing medium and small-size peptides (0.5–3.0 kDa) were more effective in stimulating the fish's immune response [26].

Regarding the impact of PAPs on antioxidant status, studies are scarce and yielded some conflicting findings. In cobia, for instance, the activity of the antioxidant enzymes CAT, superoxide dismutase (SOD), glutathione S-transferase, and glutathione peroxidase was not affected by replacing up to 60% of FM with poultry meal [54]. However, in juvenile barramundi, completely replacing FM with poultry meal led to increased LPO in serum and decreased serum glutathione peroxidase activity [14], indicating oxidative stress. Similarly, the total abolishment of FM in the FUT diet also affected the antioxidant status of the gilthead seabream, with the addition of FPH influencing this outcome.

The effects of FPH supplementation on the fish's antioxidant status are variable, with some studies reporting no effect on the antioxidant enzyme activities [19, 24, 47, 49, 58], while others report an increase [20, 21, 59, 60]. Some studies suggested that an increase in CAT and/or SOD activities indicates an enhancement of the fish's antioxidant status [20, 21]. However, the boost in the antioxidant capacity usually correlates with a reduction in LPO, as observed in fish hepatocytes conditioned with protein hydrolysates [61, 62].

Contrarily, in this study, higher CAT activity was observed in gilthead seabream fed the FUT and FUTLP diets, but LPO results did not show significant differences across treatments. Nevertheless, the similar patterns of LPO and GSH levels to CAT activity suggest that fish fed these diets experienced increased LPO, possibly due to oxidative stress triggered by the change in dietary protein source. This response aligns with other studies where low protein diets increased CAT activity, while supplementation with FPH reduced it [60]. Similarly, Tejpal et al. [16] reported decreased SOD activities in the gills and liver of pompano-fed FPH-supplemented diets, suggesting that FPHs were able to scavenge the free radicals generated during normal metabolism, thus minimizing SOD expression. Interestingly, including LPs in the FUT diet did not alleviate oxidative stress, whereas the FUTSP diet did, indicating that low MW peptides might be more effective in counteracting oxidative stress. This observation corroborates previous reports showing that low MW peptides have superior antioxidant properties [19, 26]. In fact, FPH from blue whiting whole-body has demonstrated remarkable in vitro antioxidant properties [15]. The observed antioxidant response is consistent with the immune biomarker results, suggesting that gilthead seabream able to mitigate oxidative stress also exhibited an enhanced immune response.

## 5. Conclusion

The production of FPHs, like those used in the current study, is crucial for upcycling biomass losses while adding value. Their inclusion in gilthead seabream diets has shown the potential to mitigate or even reverse the negative effects of no-FM diets on fish growth performance, immune response, and antioxidant status. Furthermore, FPHs contribute to ensuring that future diet formulations, based on ingredients that foster a circular food production system, do not increase the environmental impact of aquaculture.

Future research is needed to explore varying levels of FPH inclusion to refine these findings and develop a diet that yields similar results to those with higher levels of FM. Additionally, it will be valuable to test these and similar dietary formulations under continuous challenges (e.g., high densities) or sporadic stressors (e.g., sudden increases in water temperature, grading) to assess whether the observed enhancement in antioxidant and immune status increases fish resilience.

These research results provide important data for advancing aquafeed formulation, ultimately contributing to a more reliable, resilient, and sustainable aquaculture production.

## Figures and Tables

**Table 1:** Formulation and proximate composition of the experimental diets.

Ingredients (%)	COM	FUT	FUTLP	FUTSP
Fishmeal super prime^a^	35.00	—	—	—
Poultry meal^b^	10.00	25.00	25.00	25.00
Soy protein concentrate^c^	5.00	—	—	—
Fish protein hydrolysate^d^	2.50	—	—	—
Feather meal hydrolysate^e^	—	7.50	—	—
Porcine hemoglobin powder^f^	—	2.50	—	—
FPH LP^g^	—	—	11.30	—
FPH SP^h^	—	—	—	12.80
Wheat gluten^i^	5.50	5.50	5.50	5.50
Corn gluten meal^j^	7.50	15.00	15.00	15.00
Sunflower meal^k^	—	12.00	12.00	12.00
Microbial protein biomass^l^	—	2.50	2.50	2.50
Brewer's yeast^m^	2.50	2.50	2.50	2.50
Wheat meal^n^	18.30	10.75	10.25	10.05
Fish oil^o^	6.60	6.60	6.60	6.60
Soybean oil^p^	4.60	5.80	5.30	4.00
Soy lecithin^q^	0.50	0.50	0.50	0.50
Vitamin and mineral premix^r^	1.00	1.00	1.00	1.00
Vitamin C^s^	0.05	0.05	0.05	0.05
Vitamin E^t^	0.15	0.15	0.15	0.15
Betaine HCl^u^	0.18	0.18	0.18	0.18
Antioxidant^v^	0.20	0.20	0.20	0.20
Sodium propionate^w^	0.10	0.10	0.10	0.10
Monocalcium phosphate^x^	0.30	1.35	1.35	1.35
L-Lysine^y^	—	0.60	0.30	0.30
L-Tryptophan^z^	—	—	0.10	0.10
DL-Methionine^aa^	—	0.10	—	—
L-Taurine^bb^	—	0.10	0.10	0.10
Yttrium oxide^cc^	0.02	0.02	0.02	0.02
Proximate composition (% as fed)*⁣*^*∗*^				
Dry matter	93.83	92.83	91.96	92.38
Ash	7.56	6.77	7.65	7.78
Crude protein	48.77	48.30	47.87	48.06
Crude lipids	16.40	16.00	16.17	16.09
Total phosphorus	1.31	1.23	1.31	1.25
Gross energy (MJ kg^−1^)	22.18	21.41	21.06	21.37

Abbreviations: CF, crude fat; CP, crude protein; FPH, fish protein hydrolysate; LPs, large peptides; SPs, small peptides.

^a^Fishmeal: 66.3% CP, 11.5% CF; Pesquera Diamante, Peru.

^b^Poultry meal: 64.2% CP, 12.5% CF; SAVINOR UTS, Portugal.

^c^Soycomil P: 62.2% CP, 0.7% CF; ADM, The Netherlands.

^d^CPSP 90 : 82.6% CP, 9.6% CF; Sopropêche, France.

^e^Feather meal hydrolysate: 88.8% CP, 1.6% CF; Empro Europe NV, The Netherlands.

^f^Porcine hemoglobin powder: 91.6% CP, 1.2% CF; SONAC B.V., The Netherlands.

^g^FPH from gurnard heads with a higher proportion of LPs.

^h^FPH from whole-body blue whiting with a higher proportion of SPs.

^i^VITAL: 80.4% CP, 5.8% CF; Roquette, France.

^j^Corn gluten meal: 61.2% CP, 5.2% CF; COPAM, Portugal.

^k^Sunflower meal: 42.9% CP, 3.8% CF; AGP Slovakia, s.r.o., Slovakia.

^l^AminoPro NT70 (*C. glutamicum*): 74.1% CP, 3.1% CF; Mazzoleni S.p.A, Italy.

^m^Brewer's yeast: 38.9% CP, 4.5% CF; PREMIX Lda., Portugal.

^n^Wheat meal: 11.7% CP, 1.6% CF; Molisur, Spain.

^o^Sopropêche, France.

^p^J.C. Coimbra Lda., Portugal.

^q^Lecico P700IPM; LECICO GmbH, Germany.

^r^Premix Lda., Portugal: Vitamins (IU or mg kg^−1^ diet): DL-alpha tocoferol acetate, 100 mg; sodium menadione bisulfate, 25 mg; retinyl acetate, 20,000 IU; DL-cholecalciferol, 2000 IU; thiamin, 30 mg; riboflavin, 30 mg; pyridoxine, 20 mg; cyanocobalamin, 0.1 mg; nicotinic acid, 200 mg; folic acid, 15 mg; ascorbic acid, 1000 mg; inositol, 500 mg; biotin, 3 mg; calcium panthotenate, 100 mg; choline chloride, 1000 mg; betaine, 500 mg. Minerals (g or mg kg^−1^ diet): cobalt carbonate, 0.65 mg; copper sulfate, 9 mg; ferric sulfate, 6 mg; potassium iodide, 0.5 mg; manganese oxide, 9.6 mg; sodium selenite, 0.01 mg; zinc sulfate, 7.5 mg; sodium chloride, 400 mg; calcium carbonate, 1.86 g; excipient wheat middling.

^s^ROVIMIX stay C35, vitamin C 35%; DSM Nutritional Products, Switzerland.

^t^ROVIMIX E50, vitamin E 50%; DSM Nutritional Products, Switzerland.

^u^Beta-Key 95%; ORFFA, The Netherlands.

^v^VERDILOX; ORFFA, The Netherlands.

^w^Disproquímica, Portugal.

^x^MCP: 22.7% P, 17.5% Ca; ALIPHOS, Belgium.

^y^L-Lysine: 99%; Ajinomoto EUROLYSINE S.A.S., France.

^z^L-Tryptophan: 98.5%; Ajinomoto EUROLYSINE S.A.S., France.

^aa^DL-Methionine: 99%; ADISSEO, France.

^bb^L-Taurine: 98%; ORFFA, The Netherlands.

^cc^Yttrium oxide: Höganäs Germany GmbH, Germany.

*⁣* ^*∗*^All values are reported as the mean of duplicate analysis.

**Table 2:** Apparent digestibility coefficients (ADCs) of nutrients and energy of the experimental diets.

ADC (%)	COM	FUT	FUTLP	FUTSP
Dry matter	71.6 ± 7.1^a^	53.1 ± 4.9^b^	66.8 ± 7.0^ab^	64.2 ± 6.7^ab^
Protein	92.9 ± 1.0^a^	85.8 ± 2.3^b^	92.7 ± 1.0^a^	91.4 ± 0.4^a^
Lipids	97.1 ± 0.1^a^	83.6 ± 3.3^c^	95.1 ± 1.4^ab^	93.9 ± 0.6^b^
Energy	90.6 ± 2.0^a^	76.0 ± 2.5^c^	87.6 ± 2.3^ab^	84.7 ± 0.8^b^

*Note:* Values are presented as means ± standard deviation (*n* = 3). Different superscripts within the same row indicate significant differences (*p* < 0.05) among treatments. COM, commercial diet; FUT, future diet; FUTLP or FUTSP, FUT diet containing fish protein hydrolysates with large (LPs) or small peptides (SPs).

**Table 3:** Growth and somatic indexes of gilthead seabream juveniles fed a commercial (COM) or a future (FUT) diet containing fish protein hydrolysates with large (FUTLP) or small (FUTSP) peptides for 8 weeks.

KPI	COM	FUT	FUTLP	FUTSP
Initial weight (g)	8.00 ± 0.05	7.93 ± 0.04	8.00 ± 0.03	8.01 ± 0.08
Final weight (g)	47.74 ± 8.52^a^	33.51 ± 6.66^c^	39.15 ± 7.40^b^	40.23 ± 7.50^b^
Weight gain (% IBW)	481.6 ± 21.6^a^	310.1 ± 33.3^c^	397.0 ± 11.9^b^	410.4 ± 34.2^ab^
DGI	3.01 ± 0.08^a^	2.29 ± 0.13^c^	2.73 ± 0.01^b^	2.76 ± 0.12^ab^
*K*	1.53 ± 0.12^a^	1.42 ± 0.09^c^	1.47 ± 0.12^bc^	1.50 ± 0.11^ab^
HSI (%)	1.05 ± 0.19	1.12 ± 0.17	1.09 ± 0.19	1.03 ± 0.11
PFI (%)	1.35 ± 0.55^a^	0.93 ± 0.45^b^	0.98 ± 0.56^b^	0.95 ± 0.41^b^
Survival (%)	97.4 ± 0.6	98.2 ± 1.7	96.7 ± 2.9	98.1 ± 1.7

*Note:* Values are presented as means ± standard deviation (*n* = 54 for final weight and *K*, *n* = 3 for weight gain, DGI, and survival, *n* = 36 for HSI and PFI). Different superscripts within the same row indicate significant differences (*p* < 0.05) among treatments. The absence of superscripts indicates no significant differences. *K*, condition factor.

Abbreviations: DGI, daily growth index; HSI, hepatosomatic index; IBW, initial body weight; KPI, key performance indicators; PFI, perivisceral fat index.

**Table 4:** Feed and nutrient utilization of gilthead seabream juveniles fed a commercial (COM) or a future (FUT) diet containing fish protein hydrolysates with large (FUTLP) or small (FUTSP) peptides for 8 weeks.

KPI	COM	FUT	FUTLP	FUTSP
VFI (% day^−1^)	2.43 ± 0.16	2.94 ± 0.31	2.69 ± 0.30	2.48 ± 0.09
FCR	0.93 ± 0.05^b^	1.29 ± 0.18^a^	1.07 ± 0.13^ab^	0.98 ± 0.06^b^
PER	2.21 ± 0.12^a^	1.63 ± 0.21^b^	1.97 ± 0.22^ab^	2.13 ± 0.13^a^
PR (%)	33.3 ± 1.5	25.8 ± 3.7	30.1 ± 3.7	32.7 ± 2.0
LR (%)	61.4 ± 10.7	43.7 ± 1.8	48.9 ± 1.8	57.3 ± 11.4
ER (%)	33.9 ± 3.3^a^	25.6 ± 2.6^b^	30.6 ± 2.9^ab^	35.1 ± 3.9^a^

*Note:* Values are presented as means ± standard deviation (*n* = 3). Different superscripts within the same row indicate significant differences (*p* < 0.05) among treatments. The absence of superscripts indicates no significant differences.

Abbreviations: ER, energy retention; FCR, feed conversion ratio; KPI, key performance indicators; LR, lipid retention; PER, protein efficiency ratio; PR, protein retention; VFI, daily voluntary feed intake.

**Table 5:** Whole-body composition of gilthead seabream fed a commercial (COM) or a future (FUT) diet containing fish hydrolysates with large (FUTLP) or small (FUTSP) peptides for 8 weeks.

Fish composition (% WW)	COM	FUT	FUTLP	FUTSP
Moisture	70.6 ± 0.8	70.6 ± 0.4	70.6 ± 0.5	70.1 ± 0.9
Ash	1.0 ± 0.1	1.2 ± 0.0	1.1 ± 0.1	1.2 ± 0.1
Protein	15.3 ± 0.2^b^	16.0 ± 0.1^a^	15.6 ± 0.2^b^	15.6 ± 0.1^b^
Lipids	8.8 ± 1.0	8.3 ± 0.6	8.0 ± 0.9	8.4 ± 1.2
Energy (MJ kg^−1^)	6.8 ± 0.3	6.8 ± 0.2	6.7 ± 0.3	7.1 ± 0.4

*Note:* Values are presented as means ± standard deviation (*n* = 3). Different superscripts within the same row indicate significant differences (*p* < 0.05) among treatments. The absence of superscripts indicates no significant differences (*p* > 0.05).

Abbreviation: WW, wet weight.

## Data Availability

Data supporting reported results can be found in the publicly archived dataset generated during this study at https://doi.org/10.5281/zenodo.8422237.

## Supporting Information

Supporting Information The supporting file includes Tables S1: Proximate composition and peptide size distribution of the fish protein hydrolysates; and Table S2: Amino acid composition of experimental diets.
