# Supplementary material for: Fish Protein Hydrolysates Mitigate the Adverse Effects of No-Fishmeal Diets in Gilthead Seabream Juveniles
Source: Aquac Nutr. 2025 Mar 7;2025:1352251. doi: 10.1155/anu/1352251 (PMC11991792; doi:10.1155/anu/1352251)
Supplement: Supporting Information — The supporting file includes Tables S1: Proximate composition and peptide size distribution of the fish protein hydrolysates; and Table S2: Amino acid composition of experimental diets. [file 1352251.f1.pdf]

Supplementary material:

TABLE S1: Proximate composition and peptide size distribution of the fish protein hydrolysates.

Proximate composition (%)	Fish protein hydrolysates	
	LP	SP
Moisture	5.1 ± 1.0	4.7 ± 0.3
Ash	13.9 ± 0.4	14.8 ± 1.1
Protein	71.6 ± 1.2	63.5 ± 1.3
Total lipids	8.2 ± 0.7	16.8 ± 2.0
Average molecular weight (kDa)	2.82 ± 0.35	1.43 ± 0.1
Molecular weight distribution (% of peptides)		
< 0.2 kDa	6	16
0.2 - 0.5 kDa	8	20
0.5 – 1.0 kDa	9	21
1.0 – 3.0 kDa	59	34
> 3.0 kDa	18	9

LP: protein hydrolysate from gurnard heads with a higher proportion of large peptides; SP: protein hydrolysate from whole-body blue whiting with a higher proportion of small peptides. Results represent the means ± standard deviation (*n* = 3).

TABLE S2: Amino acid composition of experimental diets.

Amino acids (% as fed)	Diets			
	COM	FUT	FUTLP	FUTSP
Arginine	2.5	2.6	2.3	2.7
Histidine	1.0	1.1	0.8	0.9
Lysine	3.0	2.5	2.4	2.7
Threonine	2.3	1.9	3.3	2.0
Isoleucine	2.0	1.8	1.9	2.0
Leucine	4.2	4.2	5.1	4.2
Valine	2.4	2.5	2.3	2.5
Methionine	1.2	1.1	1.1	1.2
Phenylalanine	2.0	2.2	2.4	2.0
Cystine	0.6	0.7	0.6	0.5
Tyrosine	2.0	2.1	2.3	2.0
Aspartic acid	4.1	3.6	3.2	3.7
Glutamic acid	8.7	8.8	8.1	8.9
Alanine	3.1	3.0	3.2	3.1
Glycine	2.7	3.0	2.4	3.0
Proline	3.7	4.2	3.8	4.0
Serine	2.1	2.1	2.0	2.0

All values are reported as the mean of duplicate analysis. COM = commercial diet; FUT = future diet; FUTLP or FUTSP = FUT diet containing fish protein hydrolysates with a higher proportion of large or small peptides.
